# Genetic dissection of quantitative epigenomic variation in yeast

**DOI:** 10.1186/1756-8935-6-S1-P102

**Published:** 2013-03-18

**Authors:** Anne-Laure Abraham, Muniyandi Nagarajan, Jean-Baptiste Veyrieras, Hélène Bottin, Lars Steinmetz, Gaël Yvert

**Affiliations:** 1 Laboratoire de Biologie Moleculaire de la Cellule, CNRS, Ecole Normale Superieure de Lyon, 46 allee d’ltalie, 69007Lyon, France

## 

Advances in epigenomics are opening the exciting perspective to associate chromatin variation to the variation of physiological traits in natural populations. Yet, inter-individual epigenomic differences may or may not result from regulatory variation encoded in the DNA. If they do, association then resumes to mapping underlying genetic variants, as for any genetic study. If they don’t, association requires a framework where the epigenetic nature of the variation is taken into account, in particular its potential reversibility. The development of population epigenomics therefore requires an assessment of the nature of epigenomic variation. As a model experimental system, we have traced quantitative variation of acetylation at Lysine 14 of Histone H3 at all nucleosomes in a segregating population of *S. cerevisiae*. Treating acetylation of every nucleosome as a quantitative trait allowed the identification of hundreds of genetic loci underlying epigenomic divergence either locally (in *cis*) or distantly (in *trans*), and this genetic control overlapped only partially with the genetic control of gene expression. We also estimated the stability of epigenetic differences across environmentally induced reprogramming. Strikingly, ‘labile’ and ‘persistent’ nucleosomal variations were associated with poor and strong genetic control, respectively. The study reveals the dual nature of natural chromatin epi-polymorphisms (DNA-encoded vs. not), providing a basis for the development of population epigenomics. Supported by grant SiGHT nº281359 from the E.U.

